# Lamina-associated polypeptide (LAP)2α and nucleoplasmic lamins in adult stem cell regulation and disease^☆^

**DOI:** 10.1016/j.semcdb.2013.12.009

**Published:** 2014-05

**Authors:** Kevin Gesson, Sandra Vidak, Roland Foisner

**Affiliations:** Max F. Perutz Laboratories, Department of Medical Biochemistry, Medical University Vienna, Dr. Bohr-Gasse 9, 1030 Vienna, Austria

**Keywords:** ASC, (somatic) adult stem cell, BAF, barrier-to-autointegration factor, Dam, DNA adenine methyltransferase, DCM, dilated cardiomyopathy, EDMD, Emery Dreifuss muscular dystrophy, ESC, embryonic stem cell, FPLD, familial partial lipodystrophy, INM, inner nuclear membrane, HGPS, Hutchinson–Gilford Progeria Syndrome, iPS, induced pluripotent stem cell, LAD, lamina-associated domain, LAP, lamina-associated polypeptide, LEM, LAP2-Emerin-Man1, LRD, lamin rich domain, MDPSC, muscle-derived stem/progenitor cells, MSC, mesenchymal stem cell, NE, nuclear envelope, pRb, retinoblastoma protein, Adult stem cells, Lamins, Laminopathies, Nuclear envelope, Nuclear envelopathies, Progeria, Self-renewal

## Abstract

A-type lamins are components of the lamina network at the nuclear envelope, which mediates nuclear stiffness and anchors chromatin to the nuclear periphery. However, A-type lamins are also found in the nuclear interior. Here we review the roles of the chromatin-associated, nucleoplasmic LEM protein, lamina-associated polypeptide 2α (LAP2α) in the regulation of A-type lamins in the nuclear interior. The lamin A/C–LAP2α complex may be involved in the regulation of the retinoblastoma protein-mediated pathway and other signaling pathways balancing proliferation and differentiation, and in the stabilization of higher-order chromatin organization throughout the nucleus. Loss of LAP2α in mice leads to selective depletion of the nucleoplasmic A-type lamin pool, promotes the proliferative stem cell phenotype of tissue progenitor cells, and delays stem cell differentiation. These findings support the hypothesis that LAP2α and nucleoplasmic lamins are regulators of adult stem cell function and tissue homeostasis. Finally, we discuss potential implications of this concept for defining the molecular disease mechanisms of lamin-linked diseases such as muscular dystrophy and premature aging syndromes.

## Highlights

• A-type lamins localize to the lamina at the nuclear periphery and throughout the nucleoplasm.
• A-type lamins in the nuclear interior bind to and depend on lamina-associated polypeptide 2α (LAP2α).
• Lamin A/C–LAP2α complexes regulate tissue progenitor cell proliferation through retinoblastoma protein-linked pathways.
• A-type lamins regulate chromatin organization and gene expression at the nuclear periphery and throughout the nucleoplasm.
• Deregulation of nucleoplasmic A-type lamins and LAP2α contributes to abnormal phenotypes in laminopathies.

## 1 Introduction

**Fig. 1:**
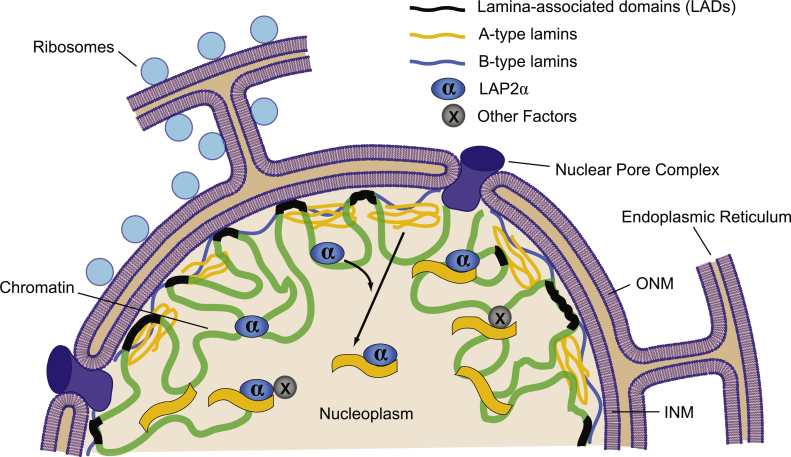
LAP2α facilitates translocation of A-type lamins to the nucleoplasm. Peripheral A-type lamins and nucleoplasmic A-type lamins, alone or in complex with LAP2α, may regulate chromatin organization.

The nuclear lamina is a proteinaceous network in metazoan cells that underlies the inner nuclear membrane (INM) and provides mechanical stability for the nuclear envelope (NE) (Fig. 1) [1–3]. It also fulfills a plethora of functions in chromatin organization, gene expression and signaling during development and tissue maintenance [4–10]. The lamina network is formed by type V intermediate filaments, the lamins [11–13], and a large number of lamin-binding proteins of the INM [14,15]. Structurally and functionally, lamins are grouped into A- and B-type lamins [16]. The main B-type lamins, lamin B1 and lamin B2 are encoded by *LMNB1* and *LMNB2*, respectively, and at least one B-type lamin is expressed in most cells throughout development. A-type lamins are encoded by the *LMNA* gene, giving rise to two major isoforms, lamin A and C, which are expressed later in development and in a differentiation-dependent manner [17]. Importantly, B-type lamins are processed post-translationally to yield a C-terminally farnesylated mature protein that is tightly associated with the INM through its hydrophobic farnesyl group. In contrast, newly synthesized pre-lamin A is also farnesylated during processing, but in a final maturation step a C-terminal peptide, including the farnesyl group, is proteolytically cleaved, producing a non-farnesylated mature lamin A [18–20]. Therefore, unlike B-type lamins, A-type lamins are less tightly linked to the INM and the lamina and are also found in a more mobile and dynamic pool throughout the nucleoplasm [21–24]. However, the regulation and specific functions of this dynamic, nucleoplasmic pool of A-type lamins are still poorly understood. Recent studies revealed evidence for exciting novel functions of this nucleoplasmic lamin pool in chromatin organization, cell signaling and cell cycle control in adult tissue stem cells (ASCs). In this review we discuss the potential functions of nucleoplasmic A-type lamins in fine-tuning the balance between proliferation and differentiation of ASCs, which is of crucial importance for tissue homeostasis. We also discuss how nucleoplasmic A-type lamins may affect the regulation of stem cell activity and how these functions may be altered in lamin-linked diseases.

## 2 Interplay between A-type lamins and LAP2α

Lamina-associated polypeptide 2 α (LAP2α) is one of six splice variants of the mammalian *LAP2* gene (originally termed *TMPO)* [25–28]. All LAP2 isoforms share the first 187 N-terminal residues [29] harboring the LAP2-Emerin-MAN1 (LEM)-domain [30], which mediates interaction with DNA in a sequence-independent manner *via* the adaptor protein barrier-to-autointegration factor (BAF) [31]. The common N-terminal LAP2 domain also contains a LEM-like motif enabling direct interaction with DNA [30,31]. Thus, all LAP2 proteins interact with chromatin by several mechanisms. The C-terminal domain of LAP2α differs considerably from that of the other LAP2 isoforms. Whereas most LAP2 isoforms, such as LAP2β, are stably anchored in the INM *via* a C-terminal transmembrane domain, LAP2α is a non-membrane protein uniformly distributed throughout the nucleoplasm [32]. Furthermore, whereas the LAP2 membrane proteins primarily bind B-type lamins at the nuclear lamina [33], LAP2α’s unique C-terminal tail mediates exclusive binding to A-type lamins [22,24] and contains an additional chromosome association domain [34,35], as well as an interaction site for the cell cycle and differentiation regulator, retinoblastoma protein (pRb) [36,37].

The specific interaction of A-type lamins and LAP2α has been extensively studied by several means, including co-immunoprecipitation, cell cycle-dependent co-localization analyses and a proximity based biotin ligase assay in mammalian cells, as well as by *in vitro* solid phase overlay and pull-down experiments [22,32,38,39]. These studies revealed direct interaction of lamins A/C and LAP2α *via* their C-terminal tails [22] and a dynamic association of the proteins during the cell cycle. The nucleoplasmic lamin A/C–LAP2α complexes exist in G1 and early S-phase of proliferating cells but are absent during mitosis [32,40]. Intriguingly, LAP2α appears to be a crucial factor for the regulation and stabilization of the nucleoplasmic pool of lamin A/C and its localization in the nuclear interior (Fig. 1). In cells and epithelial tissues derived from LAP2α-deficient mice, A-type lamins localize exclusively to the nuclear lamina and are absent from the nuclear interior. Re-expression of full length LAP2α, but not of a lamin binding-defective LAP2α mutant, into LAP2α-deficient cells rescues the nucleoplasmic pool of lamin A/C [24]. Furthermore, loss of the nucleoplasmic pool of A-type lamins during myoblast differentiation correlates with the downregulation of LAP2α [41].

Therefore, LAP2α is a master regulator of the nucleoplasmic lamin A/C pool, but the mechanisms by which LAP2α affects nucleoplasmic lamins remain elusive. In G1 phase of the cell cycle, nucleoplasmic A-type lamins may originate from lamin complexes disassembled in the preceding mitosis, or may represent newly synthesized pre-lamin A, which may interact with LAP2α in the nucleoplasm only transiently, before they assemble into the nuclear lamina. The most intriguing scenario, however, is that A-type lamins are dynamically exchanged between the peripheral and the nucleopasmic pool, depending on post-translational modifications and/or the interaction of LAP2α and other factors.

## 3 Role of A-type lamins in disease

In 1999, Bonne et al. described the first mutation in the *LMNA* gene linked to autosomal dominant Emery Dreifuss muscular dystrophy (EDMD) [42]. Since then about 400 disease-linked mutations were identified in A-type lamins and in several lamin-binding proteins of the nuclear envelope.

These mutations cause a variety of diseases, collectively termed primary laminopathies for lamin A/C-linked diseases and nuclear envelopathies for diseases linked to nuclear envelope proteins They affect different tissues (striated muscle, heart, fat, bone, skin, or neuronal tissues) in isolation or in various combinations, or cause premature aging diseases, *e.g.*, Hutchinson–Gilford Progeria Syndrome (HGPS) [43–47].

Also a mutation in *LAP2α* has been linked to dilated cardiomyopathy (DCM) [39], the pathological features of which resemble those of lamin A-linked DCM. Interestingly, this DCM-causing LAP2α mutation, which leads to a single amino acid exchange in the C-terminal lamin A/C-binding domain of LAP2α was shown to impair LAP2α’s interaction with lamin A/C *in vitro* [39].

Most disease-causing mutations in the *LMNA* gene are heterozygous single point mutations in *LMNA* found throughout the gene, leading to the expression of mutant lamin A/C variants with a single amino acid exchange. In contrast, the majority of mutations linked to HGPS introduce a cryptic splice site in exon 11 of *LMNA*, causing incorrect splicing and generation of a slightly smaller pre-lamin A variant (called progerin) that cannot be cleaved in the final step of post-translational processing and therefore remains permanently farnesylated [48]. Given that A-type lamins are expressed in nearly every differentiated cell, the tissue-specific phenotypes of many laminopathies are surprising, and the molecular pathways leading to the different pathological phenotypes are still not understood. Several non-mutually exclusive disease mechanisms have been proposed to explain the tissue-specific aspects and variability of laminopathic phenotypes [49,50].

### 3.1 The mechanical model

*LMNA* mutations may disrupt the stability or assembly of lamin networks, rendering the nucleus more fragile and less resistant to mechanical stress, ultimately leading to structural damage and cell death in mechanically stressed tissues [51]. This model is supported by reports that lamin A/C-deficient fibroblasts, as well as cells derived from several laminopathy patients have abnormally shaped nuclei [52–54], and skeletal muscle from EDMD patients and mouse disease models exhibit fragmented nuclei [55,56]. Biomechanical studies showed that, unlike B-type lamins, lamins A/C are the primary contributors to nuclear mechanics [54,57]. Accordingly, lamin A/C-deficient fibroblasts show decreased nuclear and cytoskeletal mechanical stiffness, increased nuclear fragility and impaired activation of mechanosensitive genes [58–61]. Mutations in A-type lamins can also disrupt nucleo-cytoskeletal coupling, leading to a disturbance of nuclear anchorage and impaired ability to transmit intracellular forces between the cytoskeleton and nuclear interior [51,62]. Therefore, the mechanical model may best describe muscular-dystrophy laminopathies, as muscles are exposed to high physical forces. For instance, cells expressing Familial Partial Lipodystrophy (FPLD)-linked lamin A/C mutants have normal nuclear stiffness, while mutations linked to EDMD and DCM result in a loss of nuclear stability [51].

### 3.2 The gene regulation model

This model proposes that mutations in A-type lamins or their associated proteins cause dysregulation of tissue-specific genes [46]. The altered regulation of genes may be caused by the impairment of heterochromatin formation and epigenetic pathways found in many laminopathic cells and in *Lmna*^−/−^ mouse cells [63–65]. Lamins can also affect signaling and gene expression by direct interactions with transcription factors and signaling molecules. In particular, signaling pathways involved in the regulation of proliferation and differentiation have been found to be affected in laminopathies, including pRb, mitogen activated protein kinase (MAPK), Notch, transforming growth factor β (TGF-β), sterol response element binding protein-1 (SREBP-1), NF-κB and Wnt/β-catenin pathways [4,10]. In support of this notion, cells and tissues derived from EDMD and DCM mouse models and patients show upregulated MAPK signaling [66], and HGPS patient cells present defective Wnt-, Notch- and pRb signaling [67–69]. In addition, in mouse models for progeria, NF-κB signaling was constitutively hyperactivated, leading to upregulation of inflammatory cytokines [70]. Lamin A/C-deficient cells show impaired activation of mechanosensitive genes (*Egr1*, *lex1* and *Mlk1*) and decreased NF-κB signaling [59,60,71], thus potentially linking the mechanical and gene regulation disease models.

### 3.3 The stem cell model

At the cellular level, this model proposes that mutations in *LMNA* result in proliferation and differentiation defects, which may well be directly linked to the mechanic and/or gene regulation defects mentioned above. This model is based on findings that A-type lamins interact functionally with two important regulators of G1 to S phase cell cycle progression, pRb and cyclin D3 [37,72,73] (see Section 4.1). Human HGPS fibroblasts show rapid growth at early passages, but undergo premature senescence at higher passage numbers [74], and murine progeria fibroblasts also undergo premature cellular senescence [75]. Both, mesenchymal stem cells expressing progerin [76] and epidermal stem cells in skin of progeria mice [67] display impaired proliferation and/or differentiation. Loss of wild-type lamin A/C or the expression of EDMD lamin A/C mutants compromise myoblast differentiation [77,78], and overexpression of wild-type lamin A or FPLD-linked lamin A mutants affect adipocyte differentiation [79]. Lamins A/C appear to be involved also in osteoblast differentiation, as knock-down of lamin A/C caused impaired osteoblastogenesis and accelerated osteoclastogenesis in human bone marrow stromal cells [80,81].

## 4 Functions of nucleoplasmic lamin A/C–LAP2α complexes and their link to disease

To date, only a handful of studies have addressed the potential functions of nucleoplasmic A-type lamins as opposed to those of lamins at the nuclear periphery. However, many of the described functions of lamins, which intrinsically have been linked to the peripheral lamina, may partly or predominantly require their presence within the nucleus. It is plausible to assume that, similar to the lamina, nucleoplasmic lamin A/C complexes can serve as a scaffold for signaling molecules [4,82] and may contribute to chromatin organization and epigenetic regulation of genes in the nuclear interior [83]. They may even contribute to nuclear mechanics by stabilizing an intranuclear meshwork that absorbs mechanical forces evenly like a sponge [60].

As in classical lamin A/C knock-out experiments or by expression of mutant versions of lamin A/C both lamin pools are likely to be affected, it is difficult to distinguish between peripheral *versus* nucleoplasmic lamin functions. Hence, the LAP2α knock-out mice, displaying significantly reduced levels of nucleoplasmic lamins A/C, provide an experimental system to selectively study the functions of A-type lamins in the nuclear interior [24].

### 4.1 Nucleoplasmic LAP2α–lamin A/C complexes in proliferation and differentiation

Both LAP2α [36,37] and lamins A and C [84,85] bind pRb *in vitro* and *in vivo*. pRb is a major cell cycle regulator that represses the activity of E2F transcription factors and thereby inhibits cell cycle progression in a phosphorylation-dependent manner [86]. In the absence of mitogenic signals or upon differentiation, pRb is hypo-phosphorylated, binds to E2F and inhibits E2F target gene transcription, allowing cells to exit the cell cycle. In the presence of mitogenic signals, pRb is heavily phosphorylated by cyclin-dependent kinases, causing release from E2F transcription factors, activation of E2F-dependent transcription and cell cycle progression. This basic pRb cell proliferation-regulating cycle is subject to further control by many additional pathways and feedback loops, some of which may also include nucleoplasmic A-type lamins.

**Fig. 2:**
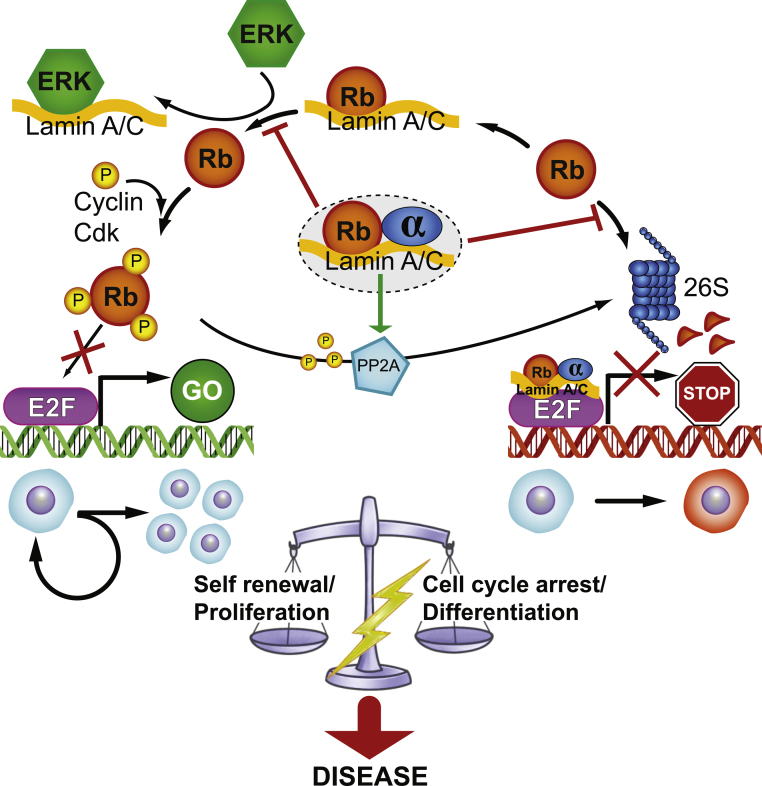
A-type lamins and LAP2α affect the cell cycle-regulating functions of pRb by several mechanisms (for details see text) balancing proliferation/self-renewal and differentiation of adult stem cells. Disease linked perturbations of lamins A/C and/or LAP2α may result in an imbalance between these two cell fates.

Several mechanisms have been proposed to explain how nucleoplasmic lamin A/C and LAP2α affect pRb function (Fig. 2): (i) A-type lamins may stabilize pRb protein, since pRb was degraded *via* the proteosomal pathway in lamin A/C-deficient cells [72]. (ii) A-type lamins may provide a scaffold for efficient dephosphorylation of pRb by PP2A protein phosphatase upon TGFß-induced cell cycle arrest [87]. (iii) Interaction of pRb with A-type lamins may keep pRb in its active (repressive) hypo-phosphorylated state. Upon growth stimulation, ERK kinase translocates to the nucleus and may compete with pRb for binding to lamins A/C, causing release of pRb and its efficient phosphorylation by cyclin-dependent kinases [88]. (iv) A complex of LAP2α, lamin A/C and hypo-phosphorylated pRb [37] may be involved in efficient E2F target gene repression [36].

In accordance with these mechanisms, nucleoplasmic lamins A/C and LAP2α were found to negatively affect cell cycle progression and thus enhance cell cycle arrest in tissue progenitor cells of regenerating tissues. Overexpression of LAP2α in cultured murine pre-adipocytes drives cells into cell cycle exit and initiates differentiation in the absence of hormones [36]. In contrast, loss of LAP2α impairs efficient cell cycle exit by contact inhibition in primary murine fibroblasts [24]. LAP2α-deficient myoblasts express higher levels of stemness factors compared to wild-type cells and show delayed differentiation *in vivo* [89]. Correspondingly, in LAP2α-deficient mice, the number of proliferating tissue progenitor cells was significantly increased in skin, colon, skeletal muscle, and in the hematopoietic system [24,40,89,90]. Furthermore, loss of LAP2α in lamin A/C-deficient mice, which lack wild-type lamin A but express low levels of a lamin A Δ8–11 variant in some cells and tissues [91], prolonged life span of double mutant mice from 30 to 70 days and partially rescued the muscle growth phenotype [92], probably by promoting proliferation of muscle progenitor cells. These findings suggest that nucleoplasmic lamin A/C–LAP2α complexes permit and/or promote differentiation of tissue progenitor cells and may thus be involved in tissue homeostasis by controlling the balance between proliferation and differentiation of adult stem cells as described in the following section.

### 4.2 Lamins in stem cell regulation

A-type lamins are absent or expressed at very low levels in undifferentiated embryonic stem cells (ESCs), and are upregulated only during cell differentiation [93,94]. Furthermore, upon reprogramming of somatic fibroblasts to induced pluripotent stem (iPS) cells, lamin A levels are vastly decreased. Knockdown of lamin A during reprogramming facilitates iPS cell generation, whereas overexpression inhibits the induction of pluripotency and drives differentiation [95].

While A-type lamins may be less important for initiation of ES cell differentiation, they may have important regulatory roles in somatic (adult) stem cells (ASCs). These are tissue-specific stem cells which serve as a clonogenic, self-renewing reservoir with the capability to differentiate into multiple cell lineages and are responsible for maintaining tissue homeostasis in adult organism by replenishing dying and non-functional cells [96]. Adult stem cells include hematopoietic and mesenchymal stem cells (MSCs). MSCs differentiate to committed precursor cells important for the regeneration of muscle, heart, bone, adipose, nerve and skin tissue, all of which are severely affected in different laminopathies. There is evidence that A-type lamins are important for regulating the maintenance and differentiation of both MSCs and tissue progenitor cells by influencing key signaling pathways [49,97]. Downregulation of A-type lamins or expression of HGPS lamin A variant in MSCs affects osteogenic, chondrogenic and adipogenic differentiation [76,81,98]. Furthermore, in two different HGPS mouse models, epidermal stem cells were depleted causing an inflammatory response [67,99]. Muscle-derived stem/progenitor cells (MDPSCs) from progeria mice also displayed defective proliferation and differentiation [100]. Interestingly, intraperitoneal administration of MDPSCs derived from young wild-type mice to progeroid mice leads to significant extension of lifespan, suggesting that impaired MDPSC function in progeria mice results in a reduced life expectancy.

Altogether, the findings that lamins alter adult stem cell function led to the hypothesis that at least part of the phenotypes observed in laminopathies are due to defects in stem cell-mediated tissue regeneration [49,101]. Increased turnover and abnormal differentiation of adult stem cells in laminopathies may also deplete the stem cell pool. The stem cell defect, coupled with a potentially increased mechanical sensitivity, could result in an inefficient repair of damaged tissues in HGPS and other laminopathies [101–103].

### 4.3 Impaired proliferation/differentiation pathways in laminopathies

Many mutations linked to laminopathies affect the localization of A-type lamins, either increasing or decreasing the nucleoplasmic pool of A-type lamins or leading to aggregation of mutant lamins in the nucleoplasm [104–107]. Therefore, it is conceivable that at least some of the molecular defects underlying laminopathies are linked to a misregulation of the functions of nucleoplasmic lamins A/C. Based on the role of nucleoplasmic lamins A/C and LAP2α in pRb-mediated cell cycle control (Fig. 2), it is tempting to speculate that mutations in these proteins affect pRb-mediated pathways and derail the balance between proliferation/self-renewal and differentiation of tissue progenitor cells. EDMD- or HGPS-linked lamin A mutants impair phosphorylation of pRb [41,108], which may lead to premature cell cycle exit and senescence and to the inhibition of differentiation [109,110]. In addition, pRb is downregulated in the Zinc metalloproteinase Ste24 homolog (Zmpste24)^−/−^ progeria mouse model [111], and genome-wide expression analysis identified the lamin A-pRb signaling network as a major pathway affected in HGPS [69]. Also the abnormal pRb localization in laminopathies [112] could further contribute to pRb dysregulation.

Besides its role in cell cycle control, pRb has well-established functions in the differentiation of muscle, adipose tissue, bone, and epidermis, all of which are affected in laminopathies. The pRb/MyoD pathway is the master regulator of myogenesis in skeletal muscle. pRb interacts with the myogenic transcription factor MyoD, subsequently activating MyoD-target genes and thereby initiating myoblast differentiation [113]. Thus, defects of the pRb pathway in laminopathies may not only affect cell cycle exit, but may also impair pRb's role in differentiation [103]. In line with this model, *Lmna*-deficient skeletal myocytes express lower levels of MyoD protein and consequently exhibit impaired MyoD/Rb-mediated *in vitro* myogenesis [77,78].

Besides pRb pathways, other differentiation-mediating signaling pathways were shown to be affected in mutant cells and tissues. The aberrant differentiation of MSCs ectopically expressing progerin was linked to increased Notch signaling [76]. Wild-type lamin A associates with the Notch co-activator SKIP, thereby scavenging SKIP and reducing Notch-dependent transcriptional activity. Progerin has reduced affinity for SKIP leading to an increase in SKIP availability and activation of Notch downstream effectors. Moreover, the Wnt/β-catenin pathway, which is known to promote stem cell proliferation in stem-cell niches of the intestine, bone marrow, brain, and epidermis, was found to be attenuated in HGPS mouse models, altering extracellular matrix production [68].

## 5 Lamins in chromatin organization and implication for laminopathies

Chromatin is non-randomly organized in the nucleus through formation of chromosome territories [114] and associations with the NE/nuclear lamina and possibly other structural components in the nucleus [7,115,8]. A-type lamins have long been proposed to be involved in the spatial organization of chromatin due to their ability to interact with DNA and core histones [11]. Similarly, several lamin binding proteins, including Lamin B Receptor (LBR) and the LEM proteins [15,30] have been shown to interact with chromatin. However, only recently a few studies revealed two redundant pathways tethering chromatin to the periphery, an LBR-mediated anchorage (probably involving B-type lamins) and an A-type lamin-LEM protein-dependent mechanism [116,117].

### 5.1 The role of the nuclear lamina in chromatin organization and gene expression

The implementation of the DamID method using a DNA adenine methyltransferase (Dam)-lamin B1 fusion protein led to the first genome-wide map of *in vivo* nuclear lamina–chromatin interactions [118,119]. The identified lamina-associated domains (LADs) were shown to be large-scale, yet sharply confined genomic regions of 0.1–10 Mb in size, which have transcriptional repressive features and represent gene-poor and heterochromatic regions with significant enrichments of repressive histone marks (H3K27me3, H3K9me3) [120]. This led to the concept that the nuclear periphery is an overall transcriptionally repressive environment as opposed to the transcriptionally permissive conditions in the nucleoplasm [83]. This model was supported by experiments showing that artificial tethering of genomic loci to the NE leads, at least in some cases, to their silencing [121–123]. Furthermore, genome-integrated arrays containing tissue-specific promoters were found to localize at the periphery in embryos and translocated to the nuclear center upon differentiation-dependent promoter activation [124]. Additionally, a lamin B1-DamID approach tracking lamina–chromatin interactions during differentiation of murine ESCs to neuronal precursor cells and to terminally differentiated astrocytes showed that previously stably NE-associated genes or gene clusters detach from the NE and subsequently become activated during differentiation [125]. These experiments indicated that the NE not only anchors heterochromatin, but may also actively contribute to the generation of a heterochromatic, transcriptionally silent environment. However, detachment from the NE *per se* does not necessarily trigger immediate activation, but may poise genes for later activation during terminal differentiation.

Opposing the view that NE–chromatin interactions are stable in the sense that they are inherited from mother to daughter cells, a recent study showed that in a given cell only 30% of all LADs are associated with the NE and are stochastically reshuffled after mitosis [126]. It remains to be investigated whether LADs containing tissue-specific genes are more specifically tethered to the NE during differentiation. Also, the “signature” (*i.e.*, the epigenetic and genetic profile) of chromatin mediating its association at the NE is poorly understood. A few recent studies have identified the heterochromatic histone mark, H3K9me2 [126,127], A/T rich sequences [128], or GAGA motifs [129] as important determinants for chromatin–NE tethering.

### 5.2 A-type lamins and chromatin regulation

Considering the dual location of A-type lamins at the nuclear lamina and in the nucleoplasm, as opposed to the exclusive peripheral localization of B-type lamins, it is conceivable that A-type lamins may also interact with chromatin in the nuclear interior (Fig. 1). At the NE, certain chromatin attachment regions may be common for both A- and B-type lamins, while other genomic regions may be exclusive to one or the other. In support of this, Shimi et al. found that A- and B-type lamins form distinct, but interconnected, networks at the nuclear lamina [130]. While the term “LAD” was originally coined for lamin B1–chromatin interactions, it appears to be extendable toward the association of lamin A with chromosomes at the NE. Genomic DamID maps for lamin A–Dam fusion proteins in human and murine cells are very similar to the genomic lamin B1 DamID maps, and constitutive NE–chromatin associations (cLADs) are highly conserved across species and cell types [128]. In addition to regions generally not associated with the lamina (constitutive inter-LADs), certain regions were found to facultatively interact with A- and B-type lamins in the course of lineage commitment, and do so with a potential preference for one or the other lamin type [128].

A recent study by Kubben et al. [131] shed light on the *in vivo* chromatin interactions of wildtype *versus* mutant lamin A linked to HGPS (progerin). In a genome-wide analysis of promoter interactions, they showed a preference of A-type lamins for binding to promoters of silent or lowly expressed genes preferentially located at the nuclear periphery. Progerin–promoter interactions overlapped to a large extent with that of wild-type lamin A, but a few progerin-specific (inherently silent) gene promoters were identified. This extensive overlap of wildtype lamin A and progerin promoter interactions is surprising, given that progerin appears to have reduced binding affinity to DNA and H3K27 trimethylated histones [132]. While the former study used overexpressed lamin A and progerin, genome interaction mapping of endogenous lamin A and progerin revealed global changes in the heterochromatic mark H3K27me3, which appeared to be lost in gene-poor regions in the mutant cells and appeared stronger in gene-rich regions, resulting in the detachment from the nuclear lamina of gene-poor heterochromatic regions [133].

Another recent study on genome-wide lamin A/C-promoter association during adipogenic differentiation identified lamin-rich domains (LRDs) throughout the genome in a manner consistent with lamin A/C–promoter interactions not being restricted to the nuclear periphery [134]. Complementary to previous findings, this study showed that binding of lamin A/C fine-tunes target gene regulation depending on the local chromatin environment in specific promoter subregions. The results indicate that lamin A/C association with promoters *per se* does not inhibit transcription, but additionally requires certain repressive histone marks in sub-promoter regions; conversely, loss of lamin A/C from promoters is a prerequisite, but not sufficient for transcriptional activation.

LAP2α is a potential candidate for mediating the interaction of nucleoplasmic lamins A/C with chromatin. LAP2α possesses a LEM and LEM-like domain [30], and thereby binds to chromatin in a sequence – independent manner. In human cells, LAP2α was found to interact with genomic DNA in a highly dynamic manner and to affect the chromatin-binding behavior of the high-mobility group N protein 5 (HMGN5) [135]. Downregulation of LAP2α led to the redistribution of HMGN5-targeted chromatin sites. Based on these findings it is tempting to speculate that LAP2α could also affect the chromatin interaction of other proteins in the nuclear interior, such as nucleoplasmic lamin A/C.

In conclusion, A-type lamins not only ensure structural integrity of (metazoan) nuclei, but also maintain the balance between differentiation and proliferation. In part, these activities may rely on lamin A/C-binding proteins, such as LAP2α. The potential effects of laminopathy-linked lamin mutants on chromatin organization may alter gene expression by destabilization of higher-order chromatin structure and/or interference with the binding of A-type lamins to other proteins. These perturbations may particularly affect stem cell homeostasis.
